# Downscaling Land Surface Temperature in Complex Regions by Using Multiple Scale Factors with Adaptive Thresholds

**DOI:** 10.3390/s17040744

**Published:** 2017-04-01

**Authors:** Yingbao Yang, Xiaolong Li, Xin Pan, Yong Zhang, Chen Cao

**Affiliations:** 1 School of Earth Science and Engineering, Hohai University, 8 Buddha City West Road, Nanjing 210098, China; yyb@hhu.edu.cn (Y.Y.); lxljnnj@163.com (X.L.); caochenalan@163.com (C.C.); 2 College of Natural Resources and Environment, Chizhou University, No.199 Muzhi Road, Chizhou 247000, China; hhuzhangyong@163.com

**Keywords:** land surface temperature, downscaling, adaptive threshold, multi-scale factors

## Abstract

Many downscaling algorithms have been proposed to address the issue of coarse-resolution land surface temperature (LST) derived from available satellite-borne sensors. However, few studies have focused on improving LST downscaling in urban areas with several mixed surface types. In this study, LST was downscaled by a multiple linear regression model between LST and multiple scale factors in mixed areas with three or four surface types. The correlation coefficients (CCs) between LST and the scale factors were used to assess the importance of the scale factors within a moving window. CC thresholds determined which factors participated in the fitting of the regression equation. The proposed downscaling approach, which involves an adaptive selection of the scale factors, was evaluated using the LST derived from four Landsat 8 thermal imageries of Nanjing City in different seasons. Results of the visual and quantitative analyses show that the proposed approach achieves relatively satisfactory downscaling results on 11 August, with coefficient of determination and root-mean-square error of 0.87 and 1.13 °C, respectively. Relative to other approaches, our approach shows the similar accuracy and the availability in all seasons. The best (worst) availability occurred in the region of vegetation (water). Thus, the approach is an efficient and reliable LST downscaling method. Future tasks include reliable LST downscaling in challenging regions and the application of our model in middle and low spatial resolutions.

## 1. Introduction

As an important parameter for characterizing the balance of surface energy, land surface temperature (LST) serves a key function in biophysical–chemical processes [1] and has been widely used in common applications, such as soil moisture estimation [2,3], forest fire detection [4], and urban heat environment monitoring [5,6,7]. Thermal infrared remote sensing (TIRS) can detect surface temperature and describe the spatial differences and diversity in LST [8] dynamically and macroscopically. A large amount of thermal remote-sensing data, including those from NOAA/AVHRR, Landsat TM/ETM+, MODIS, ASTER, and Landsat 8 TIRS, have been used to retrieve LST. Landsat satellites are frequently used in LST retrieval because of their high spatial resolution and wide availability of the data to the public, however, the LST retrieved from Landsat data is usually mixed with pixel temperature. Moreover, urban surfaces are characterized by high heterogeneity [5]. In this case, the LST retrieved from satellite-borne sensors has an insufficient spatial resolution for some urban applications. Downscaling may be applied to enhance the spatial resolution of thermal images with relatively low resolution [9].

Also known as TIRS image sharpening or scale decomposition [10], LST downscaling used to be applied at the digital number (DN) level [11] or in the fusion of images [12] before 1994. Both the DN and surface temperature/radiance (Ts/Rn) levels existed simultaneously between 1995 and 2004, and Ts/Rn has prevailed since 2005 [13,14]. Most LST downscaling methods applied at the Ts/Rn level use either statistical regressions [10,11,12,13,14,15,16,17,18] or the modulation-based technique [10]. Statistical regressions connect LST with scale factors that are extracted from high-resolution, visible, near-infrared, or short-wavelength infrared bands through statistical correlations, which are often used because of their ease of use and acceptable downscaling accuracy. The most common statistics-based downscaling algorithms include the DisTrad method [15], the TsHARP algorithm [16,17,18], the PBIM algorithm [19], the EM algorithm [20], and the HUTS algorithm [21]. Statistical regressions can be either linear or non-linear, and linear regression formulas are known for their easy implementation and application. Tom et al. [22] pioneered the use of linear regression for downscaling LST, and their findings were further improved in Nishii [23]. However, linear regression formulas may not represent the nonlinear relationships between LST and scale factors. Therefore, piecewise linear and nonlinear regression models [24,25], conditional expectation models [23], co-Kriging models [26,27], and Bayesian models [28] have been established to represent the linear or nonlinear relationships between LST and one or more scale factors. When numerous scale factors exist, the increasingly complex relationships between LST and the scale factors can be effectively determined using artificial neural networks [29], genetic algorithm techniques [30], and support vector machines [31] that can incorporate multiple scale factors and identify hidden statistical patterns. However, these techniques have a limited generalizability and cannot discern a localized and clear physical relationship. Modulation-based downscaling methods establish a function of LST or thermal radiation brightness and land cover types based on the principles of thermal radiation and spectral mixture analyses [32,33]. Zhan et al. [34] proved that these methods could achieve an excellent downscaling effect.

Most of the literature on LST downscaling have focused on the application of this technique at the Ts/Rn level. Different scale factors have been applied at varying application areas [35] and selected based on the characteristics of the study area. When the area is partly covered with vegetation, several vegetation indices can be used to downscale LST effectively [17,18], including the normalized difference vegetation index (NDVI) [15], fractal vegetation index [16,17,18], vegetation dryness index [2,36,37], and soil-adjusted vegetation index [38]. For instance, the widely adopted TsHARP method [17] uses NDVI in a linear regression model to downscale LST. However, these indices are unsuitable for LST downscaling in urban areas [19,20,21,39,40]. Given that each land cover type in cities has a unique emission rate, emissivity [19,20] has been used as a major scale factor for highly heterogeneous urban areas. Small [41] found a close relationship between surface temperature and surface albedo in urban areas. Dominguez et al. [21] integrated NDVI and surface albedo to develop the HUTS algorithm. Impervious surface percentage [40,42] and pure pixel index [43] have also been used as scale factors in urban areas. Essa et al. [40] found that substituting NDVI with impervious percentage in the DisTrad algorithm would produce better downscaling results by comparing 15 different scale factors. Essa et al. [40] and Yuan and Bauer [44] also found a strong linear relationship between LST and impervious percentage regardless of the season of image acquisition. By contrast, the relationship between LST and VI changes along with the seasons [15]. For complex urban areas with varying land cover types, multiple scale factors must be integrated to achieve a high downscaling precision. Although numerous approaches for LST downscaling have been proposed in the literature, they are often constrained by remote-sensing data and surface type. Moreover, an effective selection of scale factors for those areas with three or more land cover types is yet to be achieved.

Our study proposes a multi-scale-factor downscaling method based on an adaptive threshold for those areas with complex land cover types. A detailed analysis of errors with spatial autocorrelation between the original LST image and the downscaled products is presented. The downscaled images are compared with the images obtained by other downscaling methods through visual and quantitative analyses. As its major contributions, this study uses multiple scale factors to downscale LST in mixed areas and adaptively selects the scale factors within a moving window based on the Pearson’s correlation coefficients between LST and the scale factors. The rest of this paper is organized as follows: Section 2 discusses the proposed method. Section 3 presents the study area and data. Section 4 evaluates the downscaling results. Section 5 discusses the findings. Section 6 concludes the paper.

## 2. Methods

### 2.1. Downscaling Methods

LST can be retrieved using thermal infrared images with coarse spatial resolutions. Regression models between ancillary environmental predictors and LST have been widely established to enhance LST resolution. If the relationships between LST and the predictors do not change with the variation in the spatial resolution, a detailed LST with a high resolution can be estimated by the predictors using such relationships.

In this study, four typical ancillary environmental predictors, or scale factors, were selected to establish a multiple regression model. These factors, which include (1) SAVI [45], (2) normalized multi-band drought index (NMDI) [45], (3) modified normalized difference water index (MNDWI) [38], and (4) normalized difference building index (NDBI) [39], correspond to the main land cover types in the study area, namely, vegetation, bare soil, water, and impervious surface, respectively.

The four scale factors and LST image with coarse resolution are regressed into the model given by:

(1) $$LST_{F}=aSAVI_{C}+bNMDI_{C}+cMNDWI_{C}+dNDBI{}_{C},$$

where *SAVI_C_*, *NMDI_C_*, *MNDWI_C_*, *NDBI_C_*, and *LST_F_* are the SAVI, NMDI, MNDWI, NDBI, and fitted coarse-resolution LST, respectively. The subscript “*C*” indicates the variable in the coarse resolution, and the subscript “*F*” refers to the variable fitted by others. The coefficients *a*, *b*, *c*, and *d* change with the moving window.

Owing to the sparse ground observations and the limited spatial representativeness of ground measurements, the LST retrieved using TIRS images was used as the reference of LST, considering the reliable accuracy of retrieval, which has an average bias of less than 1 °C [46]. The residual temperature (*e*) became the difference between the retrieved LST (*LST_R_*) and the *LST_F_*, as shown in Equation (1). This difference was due to the spatial variability in LST/SAVI and LST/NDBI:

(2) $$e=LST_{F}-LST_{R}$$

Therefore, from the coarse-resolution LST, the simulated LST with coarse resolution (*LST_C_*) could be estimated as

(3) $$LST_{C}=aSAVI_{C}+bNMDI_{C}+cMNDWI_{C}+dNDBI_{C}+e.$$

Owing to the scale invariance, the relationships between LST and the scale factors with coarse resolutions were applied to the four scale factors with high resolutions. Subsequently, a simulated LST with a high resolution (*LST_H_*) is obtained, which is given by:

(4) $$LST_{H}=aSAVI_{H}+bNMDI_{H}+cMNDWI_{H}+dNDBI_{H}+e,$$

where *SAVI_H_*, *NDBI_H_*, *MNDWI_H_*, and *NMDI_H_* are the SAVI, NDBI, MNDWI, and NMDI with high resolutions, respectively. For convenience, *LST_H_* (*LST_C_*) is regarded as the downscaled (simulated) LST, whereas *LST_R_* is regarded as the retrieved LST.

The given relationships were fitted by all scale factors because of mixed pixels. Nevertheless, the heterogeneity in a pixel decreases with the spatial resolution. Therefore, in the images with high resolutions, not all the scale factors were used in this study for the regression in every pixel. A multi-scale-factor downscaling approach based on adaptive threshold (MSFAT) was developed to solve this problem. Compared with other traditional approaches, the developed approach did not involve all scale factors in fitting the regression model. Furthermore, the actual cover types were considered in the selection of scale factors in our approach.

**Figure 1:**
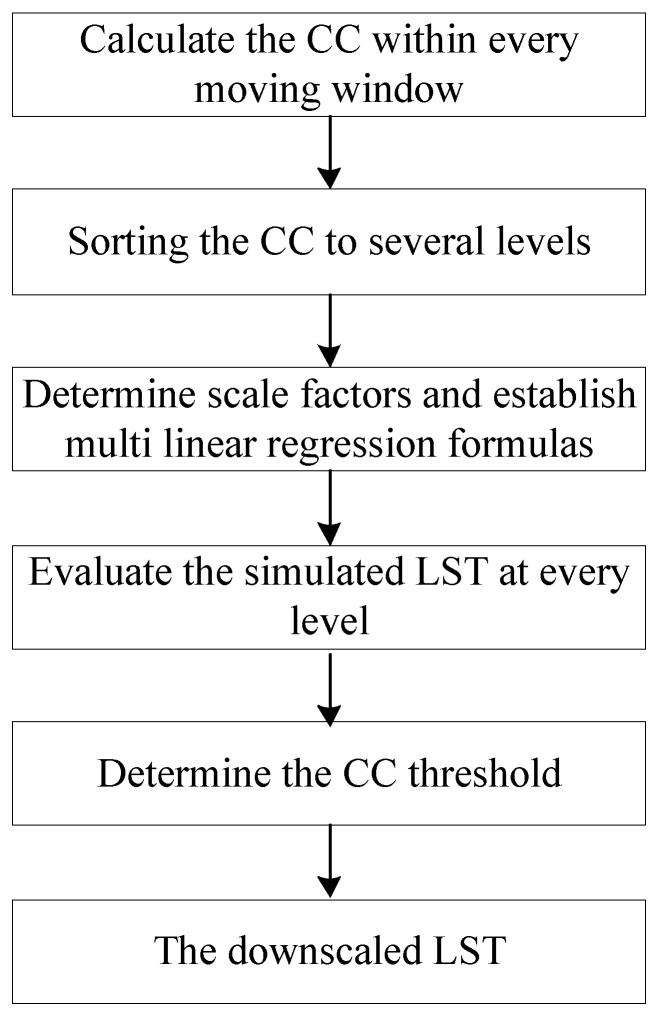
Schematic of our approach.

CCs were used to compute automatically the importance scores of the four scale factors, and CC thresholds were estimated to determine which scale factors would be involved in fitting the regression model. The estimation process of the CC threshold is as follows (Figure 1). First, the CC between each scale factor and LST was calculated within every moving window until the entire image was scanned. Second, the CCs of every scale factor were sorted into several levels (the number of windows) in ascending order. Third, at a given level, the scale factors were selected within every moving window according to the CC level, and the multiple regression model was fitted using the selected scale factors (Equation (4)). Fourth, the simulated LST with a high resolution at every level was evaluated using some evaluation measures. Fifth, the CC threshold at the optimal level for every scale factor was determined using the evaluation measures. Finally, assuming that the relationships between LST and the scale factors did not change with the scale, the pixels whose CCs were higher than the threshold values were downscaled in the corresponding multiple linear regression model (Equation (4)), whereas the LSTs of the other pixels were downscaled using the most relevant scale factors in the linear simulation. Therefore, in our downscaling method, not all land cover types were involved in the regression equation fitting, but only the scale factors of the main land cover types within a moving window were involved in the regression fitting. The correlation threshold of each scale factor was estimated to determine which scale factors should be involved in the downscaling model within a moving window.

### 2.2. Determination of the Moving Window Size

In the calculations of the regression models and CCs, the moving window (operation window) used in downscaling determines the accuracy of the estimated subpixel temperature. The moving window is also directly related to the complexity of the downscaling operations. The criteria for selecting the appropriate MWS according to the types and characteristics of the land cover in the study area should be determined.

Meanwhile, semivariance function is an important tool for understanding the spatial structure of local areas [26,27,35,47,48,49]. In our study, the semivariance function uses variable ranges to represent the spatial variations in the four scale factors in the entire region.

A total of eight values of the variable range, *R*, in the horizontal and vertical directions for the four scale factors are calculated as [50]:

(5) $$R\left(h\right)=\frac{\sum_{i=1}^{m}{\left[Z\left(x_{i}\right)-Z\left(x_{i}+h\right)\right]}^{2}}{2m},$$

where *R* is a variable range of a scale factor; *i* = 1, …, *m*, where *m* is the number of all the pixels for comparison; *x_i_* is the location of a pixel; *Z*(*x_i_*) is the value of the pixel; and *h*, which varies from 0 to 300, is the step length of curve fit. Subsequently, *R* can be determined using *h* when the semivariance fitting curve stabilizes, and *R* can be regarded as the MWS.

### 2.3. Evaluation Measures

Two measures, namely, coefficient of determination (*R*^2^) and root-mean-square error (RMSE) [19,35], were used to evaluate the downscaling effect of the MSFAT algorithm and compare the proposed algorithm with three other downscaling methods.

In the equation below, *R*^2^ is the coefficient of determination between the original and downscaled images. A high *R*^2^ indicates a satisfactory downscaling. This coefficient is given by:

(6) $$R^{2}=1-\frac{\sum {\left(LST_{S}-LST_{R}\right)}^{2}}{\sum {\left(LST_{S}-\bar{LST_{R}}\right)}^{2}},$$

where *LST_S_* is the simulated LST (Equations (3) and (4)), *LST_R_* is the retrieved LST with the same number of pixels as that of *LST_R_*, and $\bar{LST_{R}}$ is the average of *LST*_R_ in the entire image.

Meanwhile, RMSE was used to test the errors between the original LST image and the downscaled image. The calculation formula for RMSE is given by

(7) $$RMSE=\sqrt{\frac{1}{MN}\sum_{i=1}^{M}\sum_{j=1}^{N}{\left(LST_{S}-LST_{R}\right)}^{2}},$$

where *M* and *N* represent the number of rows and columns of the image, respectively.

When the threshold values of the scale factors are set too high, no scale factors can be fitted, and some pixels cannot be included in the simulation of LST. Therefore, the number of pixels in the solution to Equation (3) was used as the index of algorithm availability.

## 3. Study Area and Data

### 3.1. Study Area and Data Description

**Figure 2:**
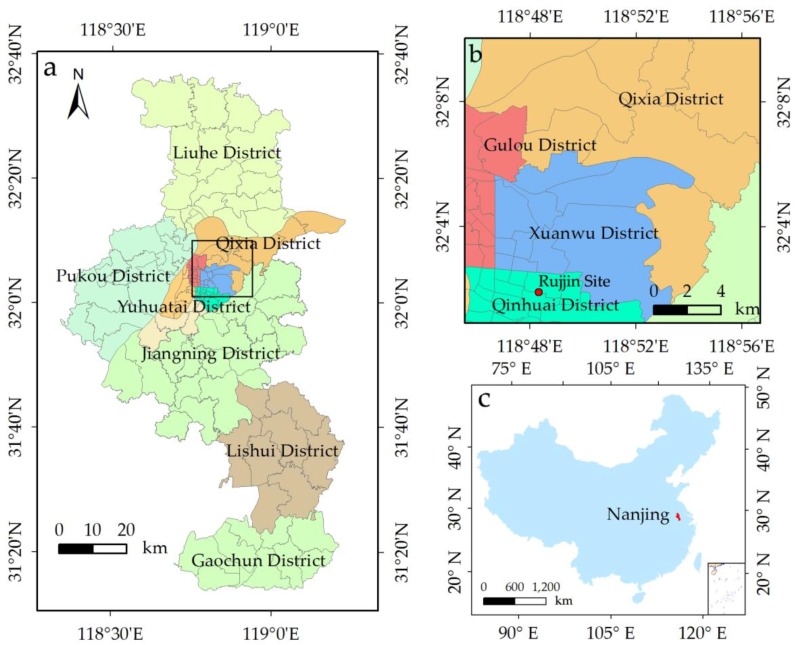
Scope of the study area in Nanjing City.

Nanjing (31°14″–32°37″ N, 118°22″–119°14″ E) is the capital of Jiangsu Province, and is in one of the largest economic zones in China, the Yangtze River Delta [51]. Nanjing covers seven districts and a total area of 6587 km^2^. It has a humid subtropical climate, which is influenced by the East Asian monsoon. The annual average rainfall and air temperature in Nanjing are 979 mm and 15.9 °C, respectively. July and August are the hottest and the most humid months, in which the average maximum air temperature is 32 °C [52]. Accordingly, LST peaks in the hot summer. The experimental area is a subset of Nanjing City (Figure 2) based on the land use and land cover map in 2010. The area, which is characterized by heterogeneous urban landscape patterns, has four main land cover types, namely, water, vegetation, bare soil (Yangtze River Beach and idle lands), and impervious surfaces.

To directly validate the downscaling LST, the hourly land surface temperature observations of Ruijin Site (118.8° E, 32.0° N) were selected. That site is a basic site of China Meteorological Administration (CMA). The chosen observation periods were 10:00 and 11:00 a.m. (Beijing hour) 11 August 2013.

The Landsat 8 Operational Land Imager (OLI) and TIRS images of Nanjing City were acquired on 11 August 2013 and then used in this study. The Landsat 8 datasets, which were provided by the United States Geological Survey, included OLI and TIRS images with 30 and 100 m spatial resolutions, respectively [53]. At the retrieval moment, the air temperature, humidity, pressure, visibility, wind direction, and wind speed were 38.0 °C, 44%, 1008 hpa, 12 km north, and 2.0 m/s, respectively. Except for the image in the summer, the other images under clear sky were also acquired on 28 March 2016, 14 October 2013, and 20 December 2014 to reveal the availability of our approach in the other seasons (spring, autumn, and winter).

**Figure 3:**
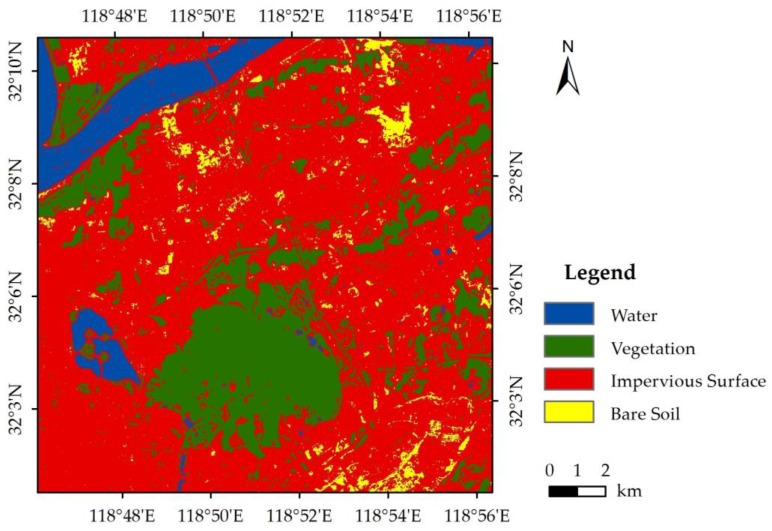
Land cover classification of the study area.

The four main land cover types were identified using the high-resolution images (Figure 3); these were then classified by maximum likelihood classification [54]. The accuracy of the classification method was evaluated by comparison with the field survey data. With a kappa coefficient of 0.915, the maximum likelihood classification thus achieved high classification accuracy. The most dominant land cover type is impervious surface area, followed by vegetation and water. No obvious law governs the spatial distribution of the four land cover types.

### 3.2. Data Processing

The data processing in this study was divided into three parts, namely, data preprocessing, downscaling processing, and simulation validation. Data preprocessing aims to unify the image resolution. Downscaling processing executes the MSFAT algorithm, and simulation validation evaluates MSFAT.

**Figure 4:**
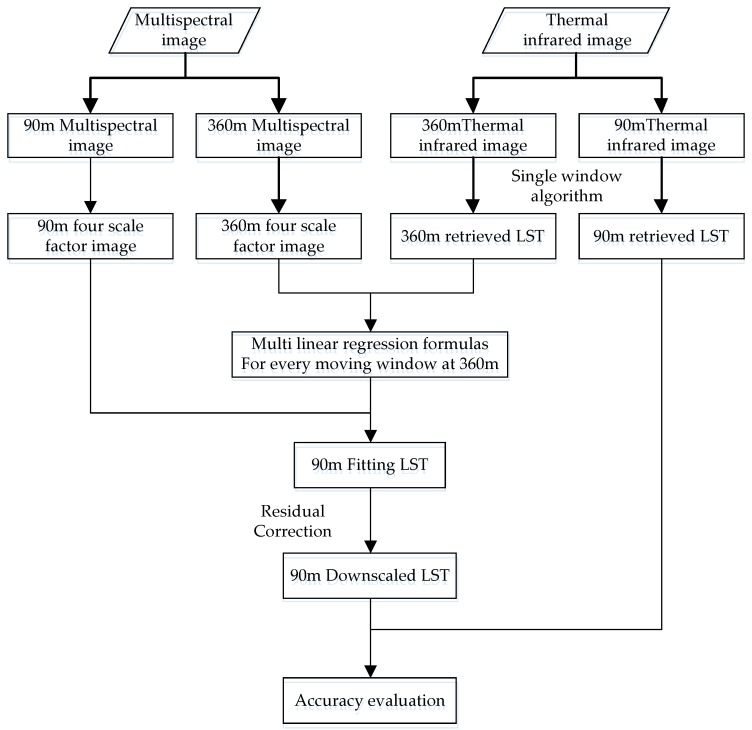
Schematic of the LST downscaling procedure.

In the data preprocessing, the OLI images were initially adjusted with the Fast Line-of-sight Atmospheric Analysis of Hypercubes (FLAASH) atmospheric correction algorithm. Owing to the unavailability of a validation reference for LST simulation, the OLI and TIRS images were upscaled to ensure that the LST simulation using Equation (4) could be validated by the LST retrieved from the TIRS images with the original resolution (Figure 4). For convenience, the TIRS images with 100 m resolution were resampled into 90 m images by the nearest neighbor method, whereas the OLI images with 30 m resolution were resampled into 90 m images by aggregation [55]. The 90 m OLI and TIRS images were also resampled by aggregation into 360 m images. The 90 m OLI and TIRS images were high-resolution images, whereas the 360 m OLI and TIRS images were coarse-resolution images. The coarse-resolution images were used to construct the relationship model in Equation (3), whereas the 90 m OLI images were used to simulate the 90 m LST in Equation (4). The 90 m retrieved LST was used to validate the 90 m simulated LST. Subsequently, the four scale factors with 90 m (360 m) resolution were then estimated from the 90 m (360 m) OLI images, whereas the LST with 90 m (360 m) resolution was retrieved from band 10 of Landsat 8 TIRS by using the generalized single-channel method in consideration of the effect of stray light [56]. Worth Noting, the final resolution of the downscaled result is 90 m.

In the downscaling process, the moving window was first adopted to explore the relationships between LST and the scale factors, and then estimated using the semivariance curve. Next, the relevant scale factors were selected, and the 360 m retrieved LST was downscaled to the 90 m simulated LST following the approach in Section 2.1.

In the simulation validation, the simulated downscaled 90 m LST image was evaluated and compared with the retrieved 90 m LST. The downscaling accuracy and the spatial distribution of the simulation error were subsequently determined.

## 4. Results

### 4.1. Spatial Distribution of LST and Scale Factors

**Figure 5:**
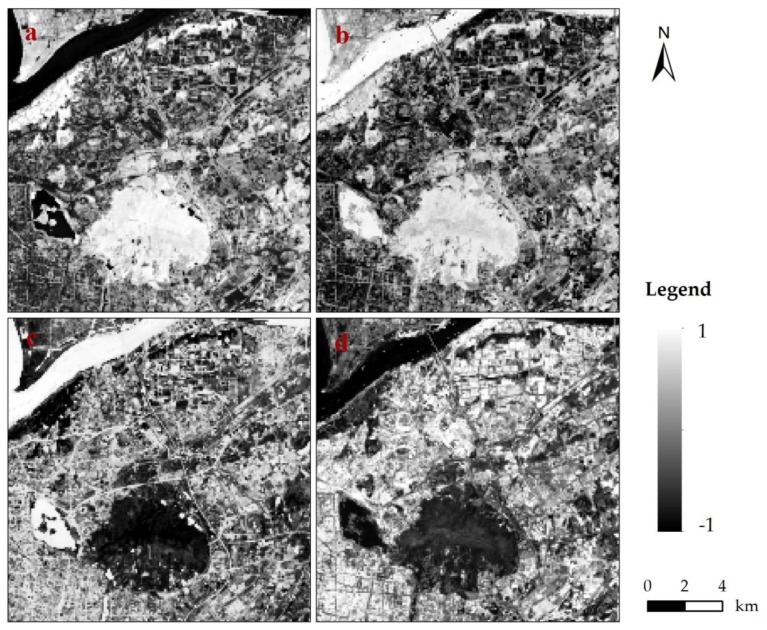
Spatial distributions of scale factors: (**a**) SAVI; (**b**) NMDI; (**c**) MNDWI; (**d**) NDBI.

The four scale factors, namely, SAVI, NMDI, MNDWI, and NDBI, were extracted from the OLI image (Figure 5). A comparison of Figure 2 and Figure 4 shows that the spatial distributions of the four scale factors and four types of land cover (vegetation, soil, water, and impervious surface) were consistent. Thus, the four scale factors can accurately characterize the four types of land cover.

The Yangzi River, flowing through the northwestern part of the study area, exhibited an MNDWI higher than 0.8, thus indicating a water area. A similar MNDWI was located in the southwest area, which corresponded to the Xuanwu Lake. In the southern part of the region, an area with SAVI of more than 1.0 was located in the Zijing Mountain with dense trees. Several building zones with NDBI of more than 0 were sporadically distributed in the northern part. Furthermore, mixed-land covers occupied the other pixels of the study area.

**Figure 6:**
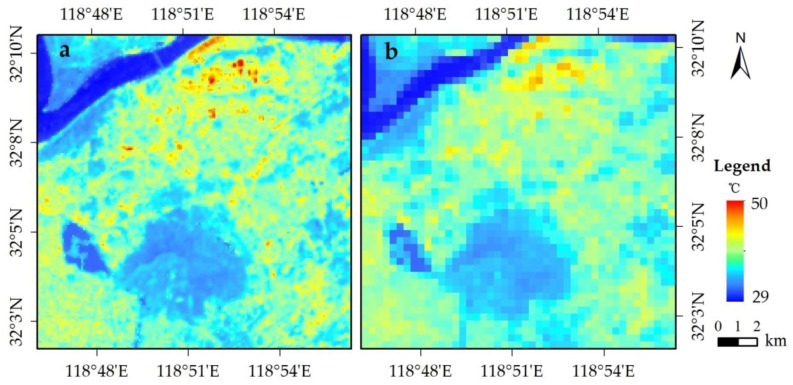
Spatial distributions of the (**a**) 90 m retrieved LST and (**b**) 360 m retrieved LST.

The distribution of LST (90 m retrieved values) is presented in Figure 6a. The average temperature in the study area was 37.2 °C. The lowest temperature (approximately 30 °C) was detected in the Yangzi River and Xuanwu Lake, which had high MNDWIs. Relatively low temperatures (32 °C–34 °C) were also recorded in the Zijing Mountain, which had a high SAVI, whereas the highest temperature (higher than 40 °C) was sporadically located in the northern industrial zones with high NDBI. The LST distribution was evidently related to the scale factors.

Figure 6b shows the 360 m retrieved LST, whose distribution is similar to that of the 90 m retrieved LST. However, detailed LST information cannot be provided in the 360 m resolution, particularly for urban areas. Thus, the downscaling approach should be applied because of the absence of high-resolution LST.

The temperature distribution matched those of the scale factors. The lowest temperature corresponded to the high MNDWIs in some western water regions (Yangzi River and Xuanwu Lake), whereas the highest temperature corresponded to the high NDBI in the northern building region. A low temperature was related to the high SAVI in the southern forest region (Zijing Mountain).

### 4.2. Downscaling Results

#### 4.2.1. Analysis of the MWS

**Figure 7:**
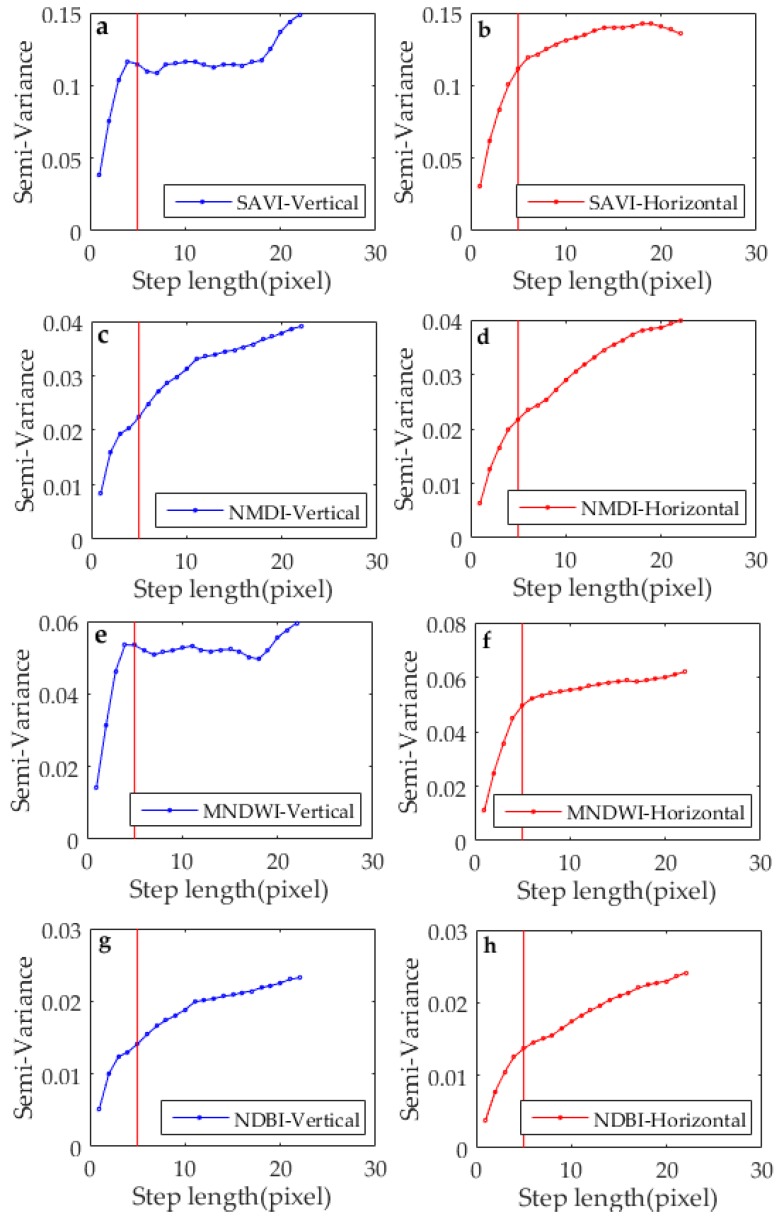
Semivariance values of scale factors in the vertical and horizontal directions, including (**a**,**b**) SAVI, (**c**,**d**) NMDI, (**e**,**f**) MNDWI, (**g**,**h**) NDBI.

The *R* value of each horizontal and vertical scaling factor was estimated using the exponential model of semivariance. As shown in Figure 7, the *R* values of the four scale factors were similar in the vertical and horizontal directions. When the step length was approximately 5 horizontally or vertically, the fitting curve of the semivariance function became stabilized, as indicated by the red line. Therefore, the average of the eight *R* values of the four land-cover types in the vertical and horizontal directions was 5. The *R* values of every scale factor in the horizontal and vertical directions were closely related to the spatial distribution characteristics. Therefore, the average values of the eight *R* values in the vertical and horizontal directions were used to determine the MWS, which was 5 × 5 pixels in this study.

#### 4.2.2. CC Threshold Value

**Figure 8:**
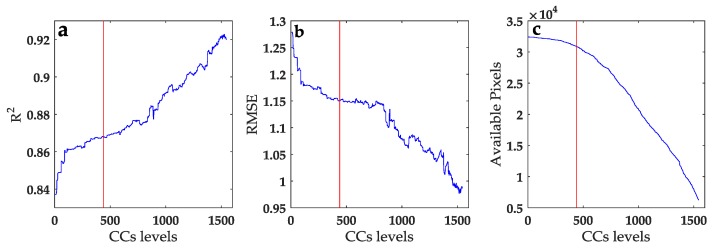
Three evaluation measures (*R*^2^, RMSE, and the number of available pixels) of the downscaled LST according to the CC levels.

**Figure 9:**
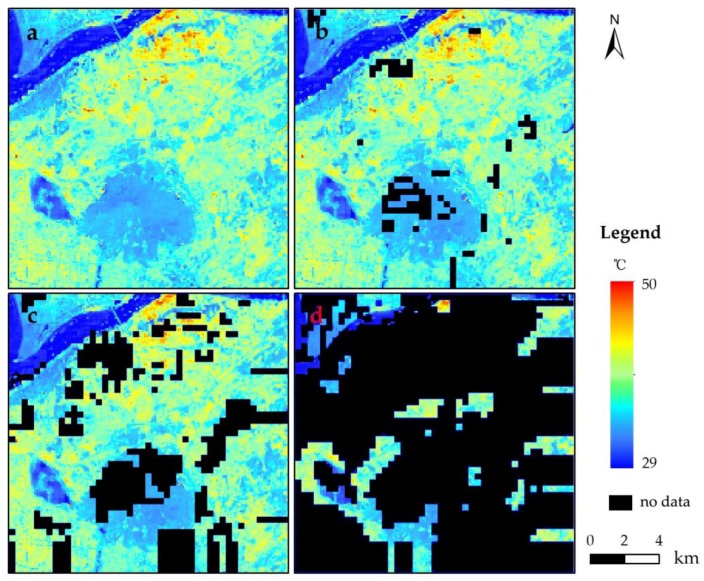
Distributions of all the available pixels in the simulated downscaled LST in the CC threshold levels (**a**) 0, (**b**) 440, (**c**) 810, and (**d**) 1550.

The CC threshold values were estimated to select the major scale factors that fit the regression model within a moving window. The CCs were sorted into 1545 levels for every scale factor from the smallest to the largest in the study area (Figure 8). The number of levels depended on the number of moving windows in the entire 360 m image. Only the scale factors with CCs higher than the corresponding CC thresholds were considered in fitting the downscaled regression model. Clearly, the higher the CC threshold is, the fewer the pixels that can be fitted. Consequently, the pixels with low CCs for every scale factor cannot be simulated. The 90 m LST was estimated using the major scale factors, which were selected on the basis of the CC thresholds. The downscaling result is shown in Figure 9.

The values of *R*^2^ and RMSE (90 m retrieved LST versus the scale factors) as well as the number of fitting pixels were used to determine the CC thresholds. Missing information (black pixels) existed because of the excessively high CCs of the scale factors in these pixels. When the CC threshold increased, *R*^2^ also increased with decreasing RMSE and decreasing number of available pixels. A low CC threshold resulted in the low accuracy of the simulated LST and a large number of available pixels, whereas a high CC threshold resulted in the high accuracy of the simulated LST and a small number of available pixels. The evaluation measures, *R*^2^, RMSE, and the number of available pixels, varied stably at level 440, where the second derivative of the curves for the measures was approximate to 0, without a dramatic variation. At this level, the average *R*^2^ was relatively large (i.e., 0.87), the RMSEs had a low average value (i.e., 1.15 °C), and more than 95% of the pixels were involved in the fitting. This meant that the accuracy was relatively unsatisfactory below this level, whereas the number of available pixels sharply decreased at this level. The fitting information of LST was lost in most of the areas with high CC thresholds (Figure 8c,d). For example, we selected four typical CC threshold levels (i.e., 0, 440, 810, and 1545) to present the discrepancies in the evaluation measures for different CC levels, because they were the beginning, stable point, dramatic variation, and final level, respectively. As shown in Table 1, when the CC threshold levels increased from 0 to 440, 810, and 1545, *R*^2^ increased from 0.84 to 0.87, 0.88, and 0.92, respectively, whereas the RMSE (number of available pixels) decreased from 1.28 °C (32,400) to 1.15 °C (30,880), 1.15 °C (25,264), and 0.99 °C (6240), respectively. Level 440 presented a balance between the downscaling accuracy and the number of available pixels.

Furthermore, the CCs at level 440 implied that the downscaled result of the MSFAT algorithm was the best. Furthermore, the optimal thresholds of SAVI, NMDI, MNDWI, and NDBI were 0.623, 0.773, 0.311, and 0.775, respectively. At these thresholds, most of the pixels can be involved in the downscaling process. In such case, the missing pixels mainly located in the Zijing Mountain, and the low CCs are probably related to the influence of uneven topography. The LST in the few noninvolved pixels had to be downscaled using most of the relevant scale factors because of the weak correlations of LST with the scale factors. Therefore, within every moving window, the related scale factors were selected, and the multiple linear regression model (Equation (3)) was established according to the respective thresholds of SAVI (i.e., 0.623), NMDI (i.e., 0.773), MNDWI (i.e., 0.311), and NDBI (i.e., 0.775).

#### 4.2.3. Downscaling Performance

**Figure 10:**
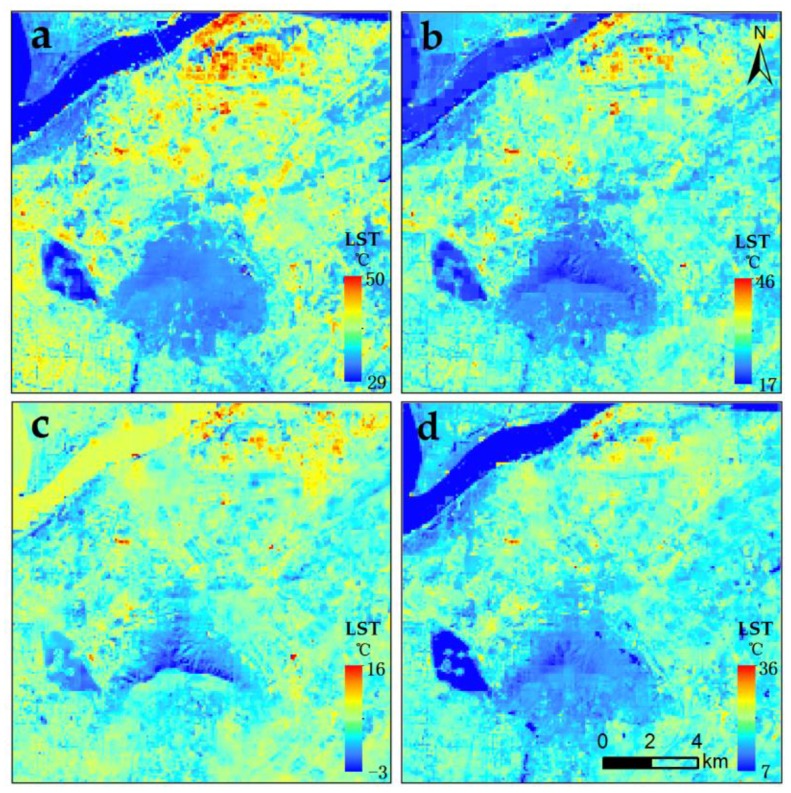
Final simulation of the 90 m downscaled LST in: (**a**) summer situation (11 August 2013); (**b**) autumn situation (14 October 2013); (**c**) winter situation (20 December 2014); (**d**) spring situation (28 March 2016).

After the fitting of the most relevant scale factors in the pixels, the final simulated result was obtained (Figure 10a). Compared with Figure 9b, Figure 10a already provides the missing information to obtain the LST in the mountain area. The average simulated temperature in the study area was 37.0 °C. A comparison of Figure 10a with Figure 5b shows that our downscaling method obviously improved the spatial resolution of the original LST image, especially in the northern region, in which high LSTs are indicated in red and yellow, corresponding to the industrial zones and the mixed areas, respectively. Our simulated LST image could also identify the bridge above the Yangzi River and the four islands in the Xuanwu Lake. The distribution of LSTs in Figure 10a is similar to those in the simulated 90 m image (Figure 9b) and retrieved 90 m image (Figure 6a), with the lowest, relatively low, and highest temperatures detected in the water, vegetation, and building areas, respectively. Therefore, our 90 m downscaled LST showed spatial reliability and provided more detailed information than the 90 m fitting LST in Figure 9b and the 360 m retrieved LST in Figure 6b.

### 4.3. Evaluation of the Downscaling Results

#### 4.3.1. Validation of the Downscaling Results

**Figure 11:**
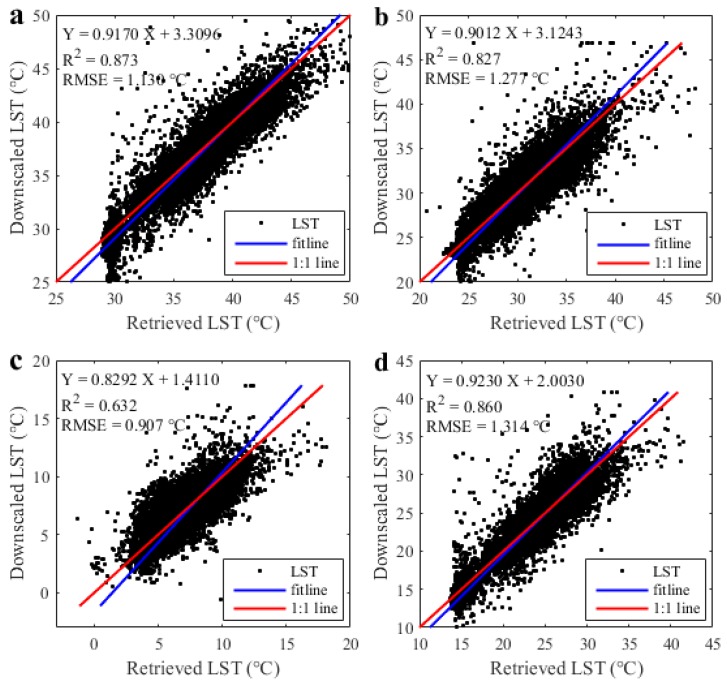
Comparison of the 90 m simulated downscaled and 90 m retrieved LST in: (**a**) summer situation (11 August 2013); (**b**) autumn situation (14 October 2013); (**c**) winter situation (20 December 2014); (**d**) spring situation (28 March 2016).

Compared with the 90 m retrieved LST, the 90 m simulated LST had a relatively satisfactory accuracy for the entire image, with pixel-average *R*^2^ and RMSE of 0.87 and 1.13 °C, respectively (Figure 11a). The pixels with LST errors of −1.0 °C–1.0 °C, −2.0 °C–−1.0 °C, 1.0 °C–2.0 °C, lower than −2.0 °C, and higher than 2.0 °C accounted for 73%, 6%, 14%, 2%, and 5% of all the pixels, respectively (Table 2). In most of the pixels, the discrepancies between the retrieved and simulated LSTs were less than 1 °C and within the scope of the retrieved accuracy [46]. Thus, reliable downscaling results appeared in most parts of the area.

**Figure 12:**
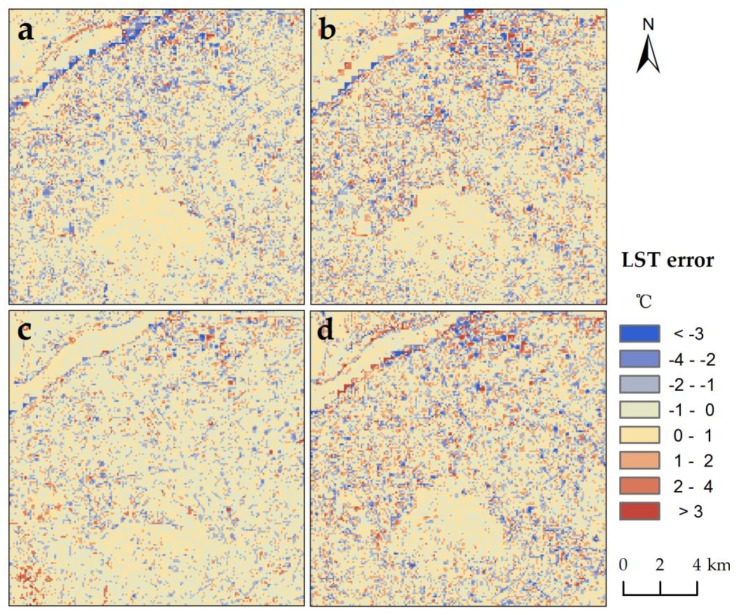
Spatial distribution of the 90 m downscaled LST minus the 90 m retrieved LST in: (**a**) summer situation (11 August 2013); (**b**) autumn situation (14 October 2013); (**c**) winter situation (20 December 2014); (**d**) spring situation (28 March 2016).

As shown in Figure 12, a systemic underestimation occurred in the region with a low temperature of approximately 30 °C, specifically in the locality near to the shores of the Yangzi River. This phenomenon may have been induced by the improper recognition of this region, such that the land–water mixed shores might have been directly mistaken for water. In addition, there were a small number of pixels with LST overestimation in the industrial zone in the northern part of the city, where dense tall factory buildings were located. This outcome might have resulted from the inaccurate estimation of land surface emissivity in the area; accordingly, the reliability of the retrieved LST was reduced [46]. In order to reveal the overall accuracy of the downscaling result, we also analyzed its accuracy depending on the different types of surfaces. The RMSE of results in the regions of water, vegetation, impervious surface, and bail soil were 1.18 °C, 0.83 °C, 1.08 °C and 1.10 °C, respectively. Our results thus demonstrate the higher accuracy in the region of vegetation than in water areas.

In addition, at the Ruijin site, the ground observation of LST was 35.02 °C. The retrieved LST and downscaling result in the pixel located at the site were 34.05 °C and 34.49 °C, respectively. Compared with ground observation, the error of downscaling result was 0.53 °C, which was within the accuracy of LST retrieval. Therefore, the direct validation also reveals the availability of our approach.

In summary, the 90 m downscaled LST proved to be reliable (with a bias of less than 1 °C) in approximately three-quarters of the area, except for the shores of the Yangzi River and the industrial zone in the northeast region. In the entire area, the pixel-average *R*^2^ and RMSE reached 0.87 and 1.13 °C, respectively.

#### 4.3.2. Comparison of Approaches

**Figure 13:**
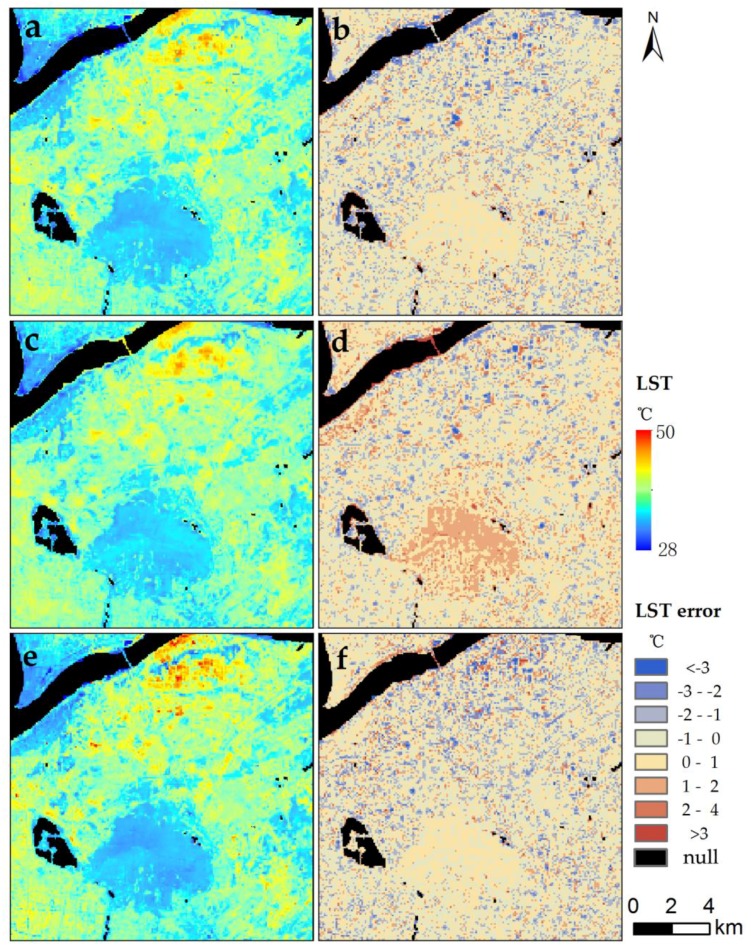
The Spatial distribution of the 90 m downscaled LST and its error for different approaches, including (**a**,**b**) DisTrad, (**c**,**d**) TsHARP and (**e**,**f**) MSFAT.

As shown in Figure 13, all downscaling methods obviously improved the spatial resolution of the original LST image (Figure 6b). Some detailed information within the same land cover was found in the downscaled images (Figure 7a,c,e); in comparison, the same cannot be found in the original image (Figure 5b). The downscaled LST images can maintain the thermal characteristics and spatial distribution characteristics of the original LST image. Relative to the 90 m retrieved LST, regardless of the water area, the downscaling result of DisTrad and TsHARP approach had a *R*^2^ (RMSE) of 0.86 (1.01 °C) and 0.82 (1.14 °C), when the value was 0.85 (1.04 °C) for our approach. Hence, the accuracy of our approach proved to be better than that of TsHARP approach, and it is also similar to that of DisTrad approach.

In detail, most errors of three methods ranged from −1 °C to 1 °C. Most of the errors were less than 1 °C for MSFAT algorithm, whereas there were more errors greater than 1 °C for the TsHARP method (Table 3). DisTrad method had the least errors of more than 3 °C but less errors ranging from −1 °C to 1 °C compared with MSFAT. The accuracy of our approach ranged between those of the DisTrad and TsHARP approaches in the regions of vegetation and impervious surface. The accuracies of these three approaches are similar in the region of bail soil. Worth noting, our approach can downscale the LST in water area, whereas the two other approaches have no ability of downscaling in the water area.

### 4.4. Availability of MSFAT in Different Seasons

Except for the situation in the summer, the downscaling results of MSFAT algorithm in the other three seasons are shown in Figure 10. Compared with the 90 m retrieved LST, the 90 m simulated LST had a relatively satisfactory accuracy for the entire image, with pixel-average *R*^2^ (RMSE) of 0.86 (1.31 °C), 0.83 (1.28 °C), and 0.63 (0.91 °C) in the spring, autumn, and winter, respectively (Figure 11). Meanwhile, the *R*^2^ and RMSE were 0.87 and 1.13 °C, respectively, in the summer. Obviously, MSFAT algorithm had the best downscaling capability in summer than in the three other seasons. In comparison, winter seemed to be incompatible with our proposed approach. This phenomenon is probably related to LST. Higher LST is always accompanied by better downscaling capability. When LST is too low, the ice and snow on the surface generally affect the scale factors and reduce the accuracy of our approach to some degree.

In detail, the accuracy of our approach was best in the region of vegetation in all seasons with RMSE of 0.73 °C–0.94 °C (Figure 12). In the region of water, the *R*^2^ (the result of our approach vs. the retrieved LST) in the winter (0.78) was obviously higher than those in other seasons (0.22–0.32), which may be related to the greater number of pure pixels in the regions of dried-up water shores. The opposite situation for RMSE appeared in the regions of impervious surface and bail soil.

Generally, the MSFAT algorithm can be applied in all seasons, especially in the summer. However, more careful application in the winter is required. Meanwhile, the best (worst) availability occurred in the region of vegetation (water) in all seasons.

## 5. Discussion

LST information derived from TIR images is essential in ecology, meteorology, and hydrology research [57,58,59]. Many applications in urban ecology require high-resolution TIR remote sensing data, and downscaling facilitates the acquisition of such data [60,61]. The primary objective of this study is to develop an adaptive selection approach for the relevant scale factors to downscale the LST maps in heterogeneous regions. In view of this objective, the usefulness of the MSFAT should be evaluated. The results of the Landsat 8 downscaling experiments indicated the effectiveness of this method. In this study, fine-scale predicted LSTs obtained by the MSFAT algorithm are compared with the retrieved LST from the original TIRS images.

The evaluation results showed relatively satisfactory RMSE and *R*^2^ statistics. Unlike in other linear regression approaches to LST downscaling, only the scale factors with CCs higher than the thresholds are involved, and the appropriate scale factors are selected adaptively in our regression. In other approaches, all the scale factors are considered without setting thresholds. As a result, all scale factors within each moving window are all included in the regression model [62,63]. A poor downscaling effect is normally attained in the pixel of a single/few land cover types if all scale factors are involved in the regression. However, in other approaches, only a single scale factor is considered; as a result, the mixed areas with numerous types of land cover cannot attain a satisfactory downscaling effect [16,17,18]. Therefore, the MSFAT algorithm has the advantages of multiple scale factors, adaptive selection of factors, and satisfactory accuracy.

However, our algorithm also has some limitations in LST downscaling in urban areas, especially those containing dense tall building blocks and river shores. This phenomenon in the regions of dense building blocks is partly related to the insensitivity of NDBI in these areas [45]. In reality, the unsatisfactory performance could be ascribed to the underestimation (overestimation) of land surface emissivity (temperature) estimation. Meanwhile, the limitation in the region of river shores (near the northwest water body) can be mainly ascribed to various mixed pixels existing in the inundated region. The dense tall building blocks and river shores occupy only few areas in ordinary urban regions. Thus, despite certain limitations, MSFAT remains an effective approach to rapidly and accurately downscaling LST in urban mixed areas because it uses adaptive thresholds and multi-scale factors. In addition, MSFAT is reliable in regions with uneven topography. However, in the regions with wavy terrain, other scale factors that can represent terrain characteristics may have to be integrated into the MSFAT algorithm. The incapability of fitting in the Zijing Mountain implies the limitation of MSFAT in mountainous areas. Furthermore, the edge effects in the downscaled LST image are apparent in the overlapping parts in the moving windows, which is due to different regression models existing in various moving windows. The edge effects may be reduced if the step size of the moving window is increased. Therefore, reliable LST downscaling in building and shore areas as well as in uneven regions will be our future goal.

Meteorological conditions differ significantly in different seasons, accompanying with the drastic variation of the state of the underlying surface. The vegetation grows luxuriantly in the summer, while the water freezes in the winter. Furthermore, the soil water content fluctuates in the rainy or dry season. Those variations possibly have an influence on the discrepancy of the availability of MSFAT in different seasons. Thus, the relationships of underling surface variations (meteorological conditions) and the availability of MSFAT will be researched in the future.

LST downscaling in middle and high spatial resolutions has been realized using our algorithm. However, limited by the low temporal resolution of the downscaled LST images and the influence of the clouds, the images are unsuitable for dynamic surface heat island analysis [47,63]. Thus, LST downscaling in middle and low spatial resolutions as well as high temporal resolution is necessary in estimating daily LST variation continuously [19,64]. The combination of LST downscaling in various resolutions contributes to the accurate monitoring of regional thermal environments [65].

## 6. Conclusions

This paper presents a strategy for downscaling LST in an area with various land cover types by using four scale factors, which are adaptively selected according to the CCs between LST and the scale factors within every moving window. The comparison results based on two statistical measures and visual analyses show that MSFAT achieves a satisfactory downscaling performance, regardless of whether it is used for vegetation area, impervious surface area, water body, and mixed area. The *R*^2^ and RMSE values between the 90 m downscaled result and the 90 m retrieved image are 0.87 and 1.13 °C, respectively. Except for the overestimation in the industrial zone in the northern region and the underestimation in the Yangzi River, the differences between the retrieved LST and simulated LST are less than 1 °C in approximately three-quarters of the study area. Spatially, compared with the 360 m retrieved LST, the 90 m downscaled result can present detailed LST information; furthermore, a similar distribution appears in both the 90 m downscaled and 90 m retrieved LSTs.

Compared with other algorithms that have been proven to provide high downscaling accuracy in our studies, MSFAT has the advantages of similar accuracy, availability of downscaling in water area, multiple scale factors, adaptive selection of factors, and a relatively credible downscaling performance. MSFAT also shows availability in all seasons, especially in the summer. Furthermore, the best availability occurred in the region of vegetation, while the worst one appeared in the region of water. Thus, MSFAT has considerable potential in generating useful LST information from thermal images of mixed areas with an improved spatial resolution. Furthermore, MSFAT can select the scale factors adaptively according to land cover types by using CCs. Thus, MSFAT can be applied to more LST data, such as MODIS/LST and ASTER/LST, in other seasons, and in other regions with flat terrain. In our future research, we intend to develop a method for performing reliable LST downscaling in building and shore areas as well as in uneven regions. Through such a method, we aim to reduce the edge effects and apply MSFAT in middle or low spatial resolutions.

## Figures and Tables

**Table 1:** Assessment of all the available pixels for the simulated downscaled LST in terms of *R*^2^, RMSE (°C), and the number of available pixels (NAP) in four CC threshold levels.

CC Threshold Level	0	440	810	1545
*R*^2^	0.84	0.87	0.88	0.92
RMSE	1.28	1.15	1.15	0.99
NAP	32400	30880	25264	6240

**Table 2:** Error probability of downscaled LST for our approach in all seasons.

LST Error (°C)	≤−3	−3–−2	−2–−1	−1–0	0–1	1–2	2–3	>3
Summer	1%	1%	6%	33%	40%	14%	3%	2%
Autumn	1%	3%	9%	32%	36%	13%	4%	2%
Winter	1%	1%	5%	34%	46%	11%	2%	1%
Spring	2%	2%	10%	32%	35%	14%	4%	2%

**Table 3:** Error probability of downscaled LST for three approaches in 11 August 2013.

LST Error (°C)	≤−3	−3–−2	−2–−1	−1–0	0–1	1–2	2–3	>3
DisTrad	1%	3%	14%	39%	31%	5%	1%	0%
TsHARP	1%	2%	10%	30%	36%	12%	1%	1%
MSFAT	1%	3%	13%	37%	31%	6%	1%	1%

## References

[1] Qin Z., Berliner P., Karnieli A. (2002). Micrometeorological modeling to understand the thermal anomaly in the sand dunes across the Israel–Egypt border. J. Arid Environ..

[2] Merlin O., Duchemin B., Hagolle O., Jacob F., Coudert B., Chehbouni G., Dedieu G., Garatuza J., Kerr Y. (2010). Disaggregation of MODIS surface temperature over an agricultural area using a time series of Formosat-2 images. Remote Sens. Environ..

[3] Sandholt I., Rasmussen K., Andersen J. (2002). A simple interpretation of the surface temperature/vegetation index space for assessment of surface moisture status. Remote Sens. Environ..

[4] Eckmann T., Roberts D., Still C. (2008). Using multiple endmember spectral mixture analysis to retrieve subpixel fire properties from MODIS. Remote Sens. Environ..

[5] Voogt J.A., Oke T.R. (2003). Thermal remote sensing of urban climates. Remote Sens. Environ..

[6] Zhou J., Chen Y.H., Wang J.F., Zhan W.F. (2011). Maximum Nighttime Urban Heat Island (UHI) Intensity Simulation by Integrating Remotely Sensed Data and Meteorological Observations. IEEE J-STARS.

[7] Su W., Yang G., Chen S., Yang Y. (2012). Measuring the pattern of high temperature areas in urban greenery of Nanjing City, China. Int. J. Environ. Res. Public Health.

[8] Weng Q., Lu D., Schubring J. (2004). Estimation of land surface temperature–vegetation abundance relationship for urban heat island studies. Remote Sens. Environ..

[9] Dennison P., Charoensiri K., Roberts D., Peterson S., Green R. (2006). Wildfire temperature and land cover modeling using hyperspectral data. Remote Sens. Environ..

[10] Zhan W., Chen Y., Zhou J., Wang J., Liu W., Voogt J., Zhu X., Quan J., Li J. (2013). Disaggregation of remotely sensed land surface temperature: Literature survey, taxonomy, issues, and caveats. Remote Sens. Environ..

[11] Moran M.S. (1990). Window-based technique for combining Landsat thematic mapper thermal data with higher-resolution multispectral data over agricultural lands. Photogramm. Eng. Remote Sens..

[12] Zhukov B., Oertel D., Lanzl F., Reinhackel G. (1999). Unmixing-based multisensor multiresolution image fusion. IEEE Trans. Geosci. Remote Sens..

[13] Gillespie A., Rokugawa S., Matsunaga T., Cothern J.S., Hook S., Kahle A.B. (1998). A temperature and emissivity separation algorithm for Advanced Spaceborne Thermal Emission and Reflection Radiometer (ASTER) images. IEEE Trans. Geosci. Remote Sens..

[14] Zhou J., Li J., Zhang L., Hu D., Zhan W. (2012). Intercomparison of methods for estimating land surface temperature from a Landsat-5 TM image in an arid region with low water vapour in the atmosphere. Int. J. Remote Sens..

[15] Kustas W.P., Norman J.M., Anderson M.C., French A.N. (2003). Estimating subpixel surface temperatures and energy fluxes from the vegetation index–radiometric temperature relationship. Remote Sens. Environ..

[16] Agam N., Kustas W.P., Anderson M.C., Li F., Colaizzi P.D. (2007). Utility of thermal sharpening over Texas high plains irrigated agricultural fields. J. Geophys. Res..

[17] Agam N., Kustas W.P., Anderson M.C., Li F., Neale C.M.U. (2007). A vegetation index based technique for spatial sharpening of thermal imagery. Remote Sens. Environ..

[18] Agam N., Kustas W.P., Anderson M.C., Li F., Colaizzi P.D. (2008). Utility of thermal image sharpening for monitoring field-scale evapotranspiration over rainfed and irrigated agricultural regions. Geophys. Res. Lett..

[19] Stathopoulou M., Cartalis C. (2009). Downscaling AVHRR land surface temperatures for improved surface urban heat island intensity estimation. Remote Sens. Environ..

[20] Nichol J. (2009). An emissivity modulation method for spatial enhancement of thermal satellite images in urban heat island analysis. Photogramm. Eng. Remote Sens..

[21] Dominguez A., Kleissl J., Luvall J.C., Rickman D.L. (2011). High-resolution urban thermal sharpener (HUTS). Remote Sens. Environ..

[22] Tom V.T., Carlotto M.J., Scholten D.K. (1985). Spatial sharpening of thematic mapper data using a multiband approach. Opt. Eng..

[23] Nishii R., Kusanobu S., Tanaka S. (1996). Enhancement of low spatial resolution image based on high resolution bands. IEEE Trans. Geosci. Remote Sens..

[24] Jing L., Cheng Q. (2010). A technique based on non-linear transform and multivariate analysis to merge thermal infrared data and higher-resolution multispectral data. Int. J. Remote Sens..

[25] Jeganathan C., Hamm N.A.S., Mukherjee S., Atkinson P.M., Raju P.L.N., Dadhwal V.K. (2011). Evaluating a thermal image sharpening model over a mixed agricultural landscape in India. Int. J. Appl. Earth Obs. Geoinform..

[26] Pardo-Igúzquiza E., Chica-Olmo M., Atkinson P.M. (2006). Downscaling cokriging for image sharpening. Remote Sens. Environ..

[27] Pardo-Iguzquiza E., Rodríguez-Galiano V.F., Chica-Olmo M., Atkinson P.M. (2011). Image fusion by spatially adaptive filtering using downscaling cokriging. ISPRS J. Photogramm. Remote Sens..

[28] Fasbender D., Tuia D., Bogaert P., Kanevski M. (2008). Support-based implementation of bayesian data fusion for spatial enhancement: Applications to ASTER thermal images. IEEE Geosci. Remote Sens. Lett..

[29] Mpelasoka F.S., Mullan A.B., Heerdegen R.G. (2001). New Zealand climate change information derived by multivariate statistical and artificial neural networks approaches. Int. J. Climatol..

[30] Yang M.-D., Yang Y.-F. (2004). Genetic algorithm for unsupervised classification of remote sensing imagery. Proceedings of the Imaging Processing: Algorithms and Systems III.

[31] Gualtieri J.A., Chettri S. (2000). Support Vector Machines for classification of hyperspectral data. Proceedings of the 2000 International Geoscience and Remote Sensing Symposium (IGARSS 2000).

[32] Wan Z., Dozier J. (1996). Generalized split-window algorithm for retrieving land-surface temperature from space. IEEE Trans. Geosci. Remote Sens..

[33] Wan Z., Dozier J. (1989). Land-surface temperature measurement from space: Physical principles and inverse modeling. IEEE Trans. Geosci. Remote Sens..

[34] Zhan W., Chen Y., Zhou J., Li J., Liu W. (2011). Sharpening thermal imageries: A generalized theoretical framework from an assimilation perspective. IEEE Trans. Geosci. Remote Sens..

[35] Zhan W., Chen Y., Wang J., Zhou J., Quan J., Liu W., Li J. (2012). Downscaling land surface temperatures with multi-spectral and multi-resolution images. Int. J. Appl. Earth Obs. Geoinform..

[36] Chen L., Yan G., Ren H., Li A. (2010). A modified vegetation index based algorithm for thermal imagery sharpening. Proceedings of the 2010 30th IEEE International Geoscience and Remote Sensing Symposium, IGARSS 2010.

[37] Sandholt I., Nielsen C., Stisen S. (2009). A Simple Downscaling Algorithm for Remotely Sensed Land Surface Temperature.

[38] Xu H. (2008). A new index for delineating built-up land features in satellite imagery. Int. J. Remote Sens..

[39] Zakšek K., Oštir K. (2012). Downscaling land surface temperature for urban heat island diurnal cycle analysis. Remote Sens. Environ..

[40] Essa W., Verbeiren B., van der Kwast J., Van de Voorde T., Batelaan O. (2012). Evaluation of the DisTrad thermal sharpening methodology for urban areas. Int. J. Appl. Earth Obs. Geoinform..

[41] Small C. (2006). Comparative analysis of urban reflectance and surface temperature. Remote Sens. Environ..

[42] Essa W., van der Kwast J., Verbeiren B., Batelaan O. (2013). Downscaling of thermal images over urban areas using the land surface temperature–impervious percentage relationship. Int. J. Appl. Earth Obs. Geoinform..

[43] Yang G., Pu R., Huang W., Wang J., Zhao C. (2010). A novel method to estimate subpixel temperature by fusing solar-reflective and thermal-infrared remote-sensing data with an artificial neural network. IEEE Trans. Geosci. Remote Sens..

[44] Yang G., Pu R., Zhao C., Huang W., Wang J. (2011). Estimation of subpixel land surface temperature using an endmember index based technique: A case examination on ASTER and MODIS temperature products over a heterogeneous area. Remote Sens. Environ..

[45] Yuan F., Bauer M.E. (2007). Comparison of impervious surface area and normalized difference vegetation index as indicators of surface urban heat island effects in Landsat imagery. Remote Sens. Environ..

[46] Sobrino J.A., Jiménez-Muñoz J.C., Paolini L. (2004). Land surface temperature retrieval from LANDSAT TM 5. Remote Sens. Environ..

[47] Rodriguez-Galiano V., Pardo-Iguzquiza E., Sanchez-Castillo M., Chica-Olmo M., Chica-Rivas M. (2012). Downscaling Landsat 7 ETM+ thermal imagery using land surface temperature and NDVI images. Int. J. Appl. Earth Obs. Geoinform..

[48] Hongchao M., Deren L. (2001). Enhancing group resolution of TM6 based on multi-variate regression model and semi-variogram function. Geo-Spat. Inf. Sci..

[49] Pardo-Igúzquiza E., Atkinson P.M. (2007). Modelling the semivariograms and cross-semivariograms required in downscaling cokriging by numerical convolution–deconvolution. Comput. Geosci..

[50] Zhang Y. (2015). Land Surface Temperature Inversion and Downscaling Research for Landsat 8. Master Thesis.

[51] Yang Y., Yao L. (2009). The influence of urban design factors on urban heat environment in urban residential area with remote sensing. Proceedings of the Sixth International Symposium on Multispectral Image Processing and Pattern Recognition.

[52] Tang C.-S., Shi B., Gao L., Daniels J.L., Jiang H.-T., Liu C. (2011). Urbanization effect on soil temperature in Nanjing, China. Energy Build..

[53] Roy D.P., Wulder M.A., Loveland T.R., Woodcock C.E., Allen R.G., Anderson M.C., Helder D., Irons J.R., Johnson D.M., Kennedy R. (2014). Landsat-8: Science and product vision for terrestrial global change research. Remote Sens. Environ..

[54] Erbek F.S., Özkan C., Taberner M. (2004). Comparison of maximum likelihood classification method with supervised artificial neural network algorithms for land use activities. Int. J. Remote Sens..

[55] Parker J.A., Kenyon R.V., Troxel D.E. (1983). Comparison of Interpolating Methods for Image Resampling. IEEE Trans. Med. Imaging.

[56] Jiménez-Muñoz J.C., Cristóbal J., Sobrino J.A., Sòria G., Ninyerola M., Pons X. (2009). Revision of the single-channel algorithm for land surface temperature retrieval from Landsat thermal-infrared data. IEEE Trans. Geosci. Remote Sens..

[57] Pan X., Liu Y., Fan X. (2015). Comparative Assessment of Satellite-Retrieved Surface Net Radiation: An Examination on CERES and SRB Datasets in China. Remote Sens..

[58] Pan X., Liu Y., Fan X. (2016). Satellite Retrieval of Surface Evapotranspiration with Nonparametric Approach: Accuracy Assessment over a Semiarid Region. Adv. Meteorol..

[59] Pan X., Liu Y., Yang Y., Fan X., Wang R. Estimation of evapotranspiration using nonparametric approach under all sky: Primary results and accuracy evaluations. Proceedings of the 2016 IEEE International Geoscience and Remote Sensing Symposium (IGARSS).

[60] Sismanidis P., Keramitsoglou I., Kiranoudis C., Bechtel B. (2016). Assessing the Capability of a Downscaled Urban Land Surface Temperature Time Series to Reproduce the Spatiotemporal Features of the Original Data. Remote Sens..

[61] Su W., Gu C., Yang G., Chen S., Zhen F. (2010). Measuring the impact of urban sprawl on natural landscape pattern of the Western Taihu Lake watershed, China. Landsc. Urban Plan..

[62] Liu D.S., Pu R.L. (2008). Downscaling thermal infrared radiance for subpixel land surface temperature retrieval. Sensors.

[63] Bechtel B., Zakšek K., Hoshyaripour G. (2012). Downscaling Land Surface Temperature in an Urban Area: A Case Study for Hamburg, Germany. Remote Sens..

[64] Mukherjee S., Joshi P.K., Garg R.D. (2014). A comparison of different regression models for downscaling Landsat and MODIS land surface temperature images over heterogeneous landscape. Adv. Space Res..

[65] Weng Q., Fu P., Gao F. (2014). Generating daily land surface temperature at Landsat resolution by fusing Landsat and MODIS data. Remote Sens. Environ..

